# Work-related musculoskeletal disorders among United Arab Emirates schoolteachers: an examination of physical activity

**DOI:** 10.1186/s12891-024-07256-w

**Published:** 2024-02-12

**Authors:** Hind Mahmoud Abu Zohair, Srilatha Girish, Animesh Hazari

**Affiliations:** 1 College of Health Sciences, Gulf Medical University, Ajman, United Arab Emirates; 2 Department of Community Physiotherapy, MGM Institute of Physiotherapy, Chh. Sambhajinagar, India

**Keywords:** Work related musculoskeletal disorders, Schoolteachers, Prevalence, United Arab Emirates, Physical activity

## Abstract

**Objective:**

To estimate the prevalence of work-related musculoskeletal disorders and their association with physical activity among schoolteachers in the United Arab Emirates.

**Methods:**

This observational cross-sectional study involved 209 schoolteachers (aged 20–60) with a minimum of two years of experience. Data, including demographics (gender, age), Nordic Musculoskeletal Questionnaire (NMQ) for WMSD prevalence and pain sites, and Global Physical Activity Questionnaire (GPAQ) for physical activity levels, were collected. Six schools were visited for data collection, with consent from school heads and participants. Schedules were tailored to participant availability, allowing up to three attempts for participation. Non-respondents were identified after three unsuccessful attempts.

**Results:**

Of the total 206 participants, 149 were female, while the remaining 57 were male. Age distribution analysis revealed that 18% of individuals were within the 20 to 30 years range, 26.2% fell within the 30 to 40 years, and 36.9% had 40 to 50 years age brackets. The responses were obtained in Arabic (90%) and English (10%). The study identified a high prevalence (71.4%) of work-related musculoskeletal disorders (WMSD) in UAE schoolteachers, with neck pain being the most common (74.3%). Major risk factors included age, workload, and low physical activity. The data was normally distributed, and Pearson’s Correlation test revealed weak positive correlation (r: 0.14), but statistically significant (*p* value = 0.04) between WMSD and PA, indicating that it was a contributing factor but other factors beyond PA influenced WMSD prevalence in this cohort. The findings of the study are based on certain limitations such as cross-sectional design and convenient sampling which could have potential selection bias and affect generalizability of the results.

**Conclusion:**

Findings suggest the need to promote physical activity and reduce workload for teachers, considering their age and gender. Additionally, there is a need to raise awareness regarding ergonomics and the importance of taking short breaks for stretching or physical movement to enhance the overall well-being of schoolteachers in the UAE and similar contexts. Diverse prevalence rates across different body areas underscore the necessity for individualized treatments.

## Background

Work-related Musculoskeletal Disorders (WMSD) represent a significant percentage of occupational health problems across most working populations [1]. Among the various populations, teaching professionals like schoolteachers are concurrently identified as being at very high risk of developing WMSD due to prolonged static positions, repetitive movements, and emotional stress inherent in their profession. These factors contribute to muscle strain and discomfort. Additionally, limited breaks and ergonomic challenges further exacerbate their vulnerability to WMSD. Recently, WMSD has garnered significant research interest after years of neglect [2]. A study conducted in Saudi Arabia reported significant prevalence of WMSD (87.3%) among schoolteacher [3]. The study found that the prevalence was higher among female teachers (95.3%), compared to males (81.4%) and the most prevalent site was lower back with smoking being the most significant risk factor. The study suggested that the impacts of WMSD include loss of physical function, deteriorated general health, decreased participation in social activities, insomnia, irritability, anxiety, and depression The study also emphasized the need for awareness and practice of ergonomics among teaching professionals [3].

According to the National Institute for Occupational Safety and Health (NIOSH), work related musculoskeletal disorders are a cluster of medical problems that affect the tendons, nerves, muscles and supporting tissues found throughout the body [4]. WMSD are characterized by degeneration or inflammation that affect the musculoskeletal system and is significantly influenced or worsened by work conditions resulting in pain, discomfort, and functional impairment in the joints, muscles, bones, ligaments, tendons, nerves, and blood vessels [5]. These include clinical syndromes including but not limited to tendon inflammation and related conditions (tenosynovitis) and nerve injuries (carpal tunnel syndrome). WMSD develops gradually over time due to physical and psychosocial stressors, occurring when the demands of work exceed the adaptive capacity of the musculoskeletal tissue.

The most common symptoms of WMSD include pain, numbness, tingling, aching, stiffness, or burning sensation. WMSD is also reported to cause lost work time or absenteeism, increase work restrictions, transfers to another job, or disability more than any other group of diseases, with a considerable economic toll on the individual, the organization, and society.

Several risk factors have been associated with WMSD including occupational, individual, and social factors. Among all, Physical Activity (PA) is a key factor in the prevention and control of WMSD. It has been found that the PA can counter occupational-related cognitive decline, anxiety, and depression [6]. The effects of PA depend on the frequency, and intensity. Several studies have explored the relationship between PA and WMSD in various occupational settings. One study conducted among teachers found that PA played a protective role in reducing the risk of musculoskeletal disorders, with inactive teachers having a significantly higher risk compared to their active counterparts [7]. Another study examined the relationship between WMSD and PA, reporting that specific pain areas showed varying associations. Individuals with neck pain had lower levels of PA in the active transport domain, while those with knee pain had higher levels in the work domain. Moreover, longer sitting time was associated with a higher likelihood of experiencing WMSD [6]. The occupational factors found to contribute to the risk of WMSD among teachers include long working hours, typically exceeding 40 h per week, and were significantly associated with a higher risk in their upper and lower limbs [8]. Similar to other geographical regions, schoolteachers in the United Arab Emirates (UAE) spend most of their time in sedentary or physically inactive states due to busy schedule and demand of the work. Considering the clinical significance of the condition, and paucity of data in UAE, it is important to determine the prevalence and the correlation between WMSD and PA among schoolteachers in UAE to use effective intervention strategies to manage and prevent these health problems. This could help to understand the pattern and factors associated with WMDS in UAE teaching professionals, exploring the need for awareness, combating strategies and necessary ergonomic modifications. Thus, the study aimed to address the empirical gap for WMDS among schoolteachers in UAE with following objectives:

Objective 1: To estimate the prevalence of WMSD among schoolteachers in UAE.

Objective 2: To investigate the PA-WMSD association among schoolteachers in UAE.

## Methodology

### Research design & settings

An observational cross-sectional study with convenient sampling methods was conducted where 6 schools from Abu Dhabi, Dubai, Sharjah, Ajman (4 government schools and 2 private schools) with female and male teachers participated in the study. The selection of these schools was based on the feasibility and permission from the administration to conduct the study.

### Inclusion and exclusion criteria

All schoolteachers from grade 1 to grade 12 (primary, secondary students), male and female, aged between 20 and 60 years, with a minimum of 2 years of experience, were eligible for our study. Pregnant women or participants with recent MSDs unrelated to their work were excluded from the study.

### Sample size calculation

Sample size calculation was based using estimated prevalence rate from previous studies. According to a recent study done in Hail, Saudi Arabia, the prevalence of WMSD was (87.3%) among secondary schoolteachers [3].

Thus, we calculate the sample size as follows.

*P* = 87.3%, *p* = 0.87, q = 1- *p* = 1-0.87 = 0.13, Z = 1.96, L = 0.05.

S = Z2pq/ L2.

S= (1.96)2 (0.87) (0.13)/(0.05)2 = 0.4344/0.0025 = 174

With expected 20% of non-response = 20% (174) = 35, the desired sample size was = 174 + 35 = 209

### Study procedure

The study received ethical approval from the Institutional Review Board (IRB-COHS-STD-59-APRIL-2023). Permission to approach participants was obtained from respective school heads. Subsequently, written informed consent was signed by the participants who were eligible for the study. The schedule for data collection was prepared as per the availability of participants. If a participant was unable to participate on the allocated day, two more attempts were made, after which the participant was considered as a non-respondent if they fail to attend/ participate. The study used the Nordic Musculoskeletal Questionnaire (NMQ) and Global Physical Active Questionnaire (GPAQ) questionnaire in its original form and English language. In addition, the Arabic translation of both questionnaires was done and validated by subject and language experts. It was translated by two Arabic language experts, reviewed by 2 subject experts of Arabic slang and one mastering English language. Further it was back translated by three different Arabic nonmedical expert translators and comparisons were made to ensure same wording and meaning of the questions, and finally reviewed by researchers. Response was collected and coded for data analysis. The data collection and analysis were done from April 2023 to October 2023.

### Data analysis

Descriptive statistics were used for all the study variables to assess the prevalence of WMSD among schoolteachers. Continuous variables were analyzed using means and standard deviation, while frequency distribution was used for all the categorical variables. The test of normality was conducted using the SW and KS tests, and the alpha value was more than 0.05 on both suggesting that the data was normally distributed for parametric test to be applied. Thus, to identify the association between the PA level and WMSD we used Pearson’s Correlation test as inferential statistics (SPSS v29 software).

### Non- respondents

Three participants were unable to participate on the allocated days, after which the participant was considered as a non-respondent and removed from data analysis. Since the actual sample size included 20% non-respondent, we consider that removing three participants from the data analysis would not affect the findings of the study as the calculated sample size was achieved for statistical inference.

## Results

**Fig. 1:**
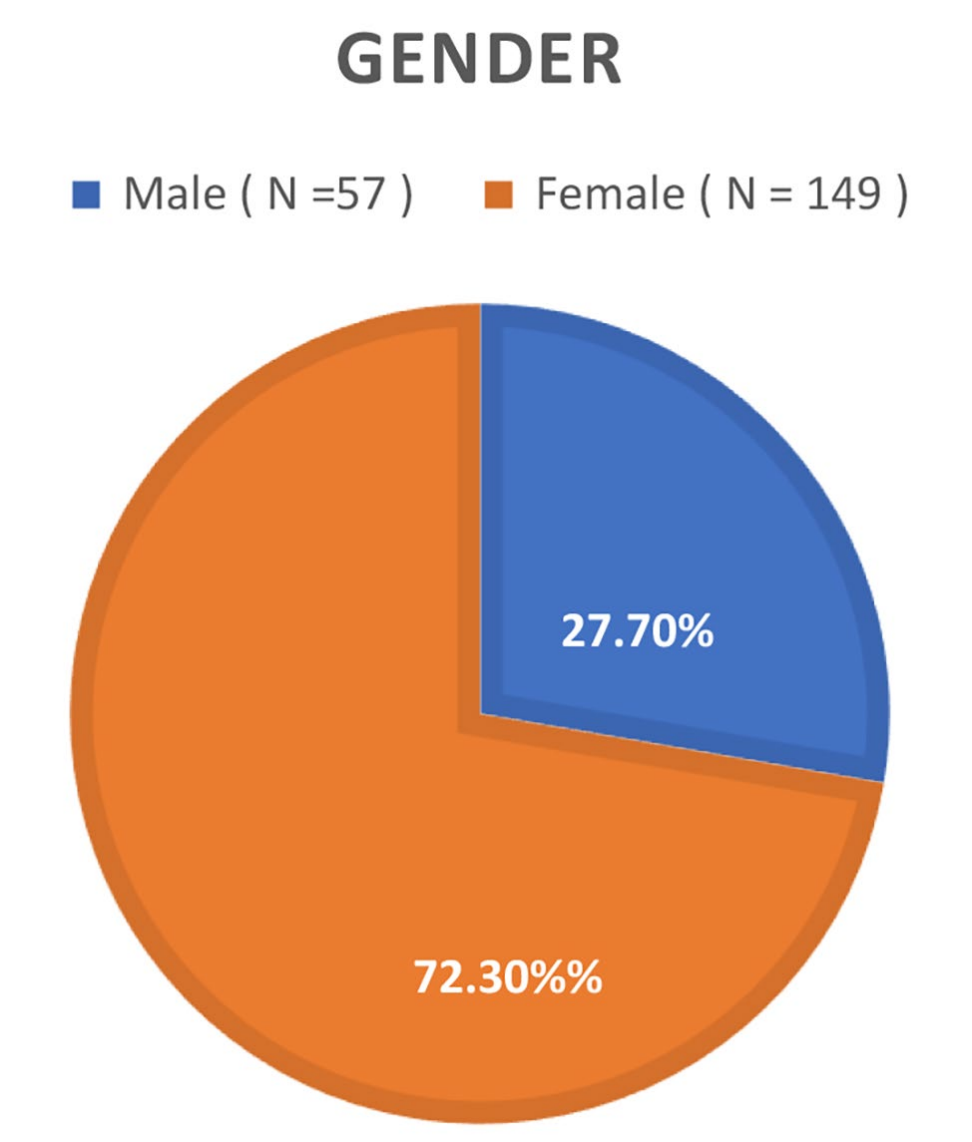
Demographic characteristics of participants (Gender)

**Fig. 2:**
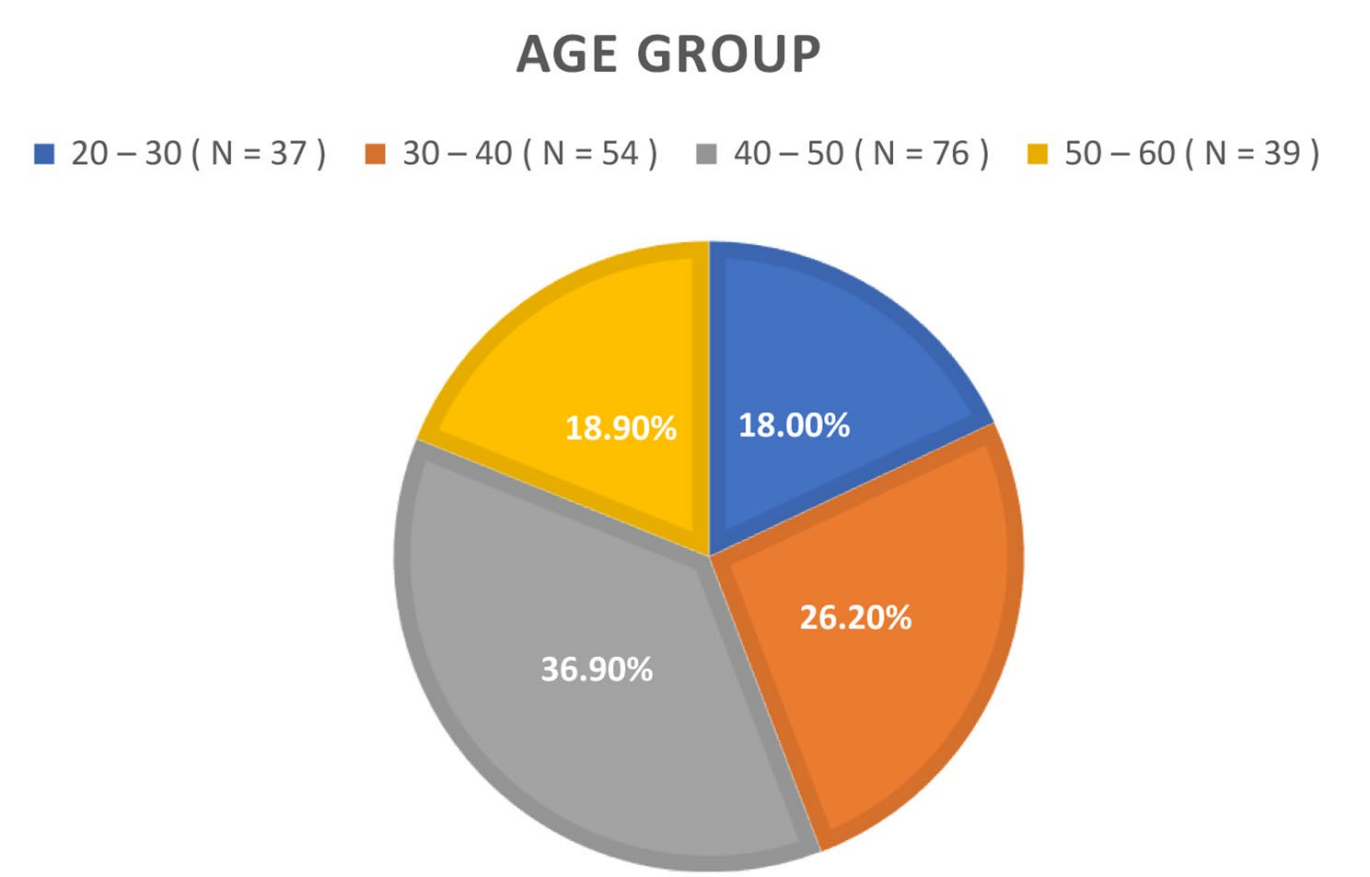
Demographic characteristics of participants (Age Groups)

**Fig. 3:**
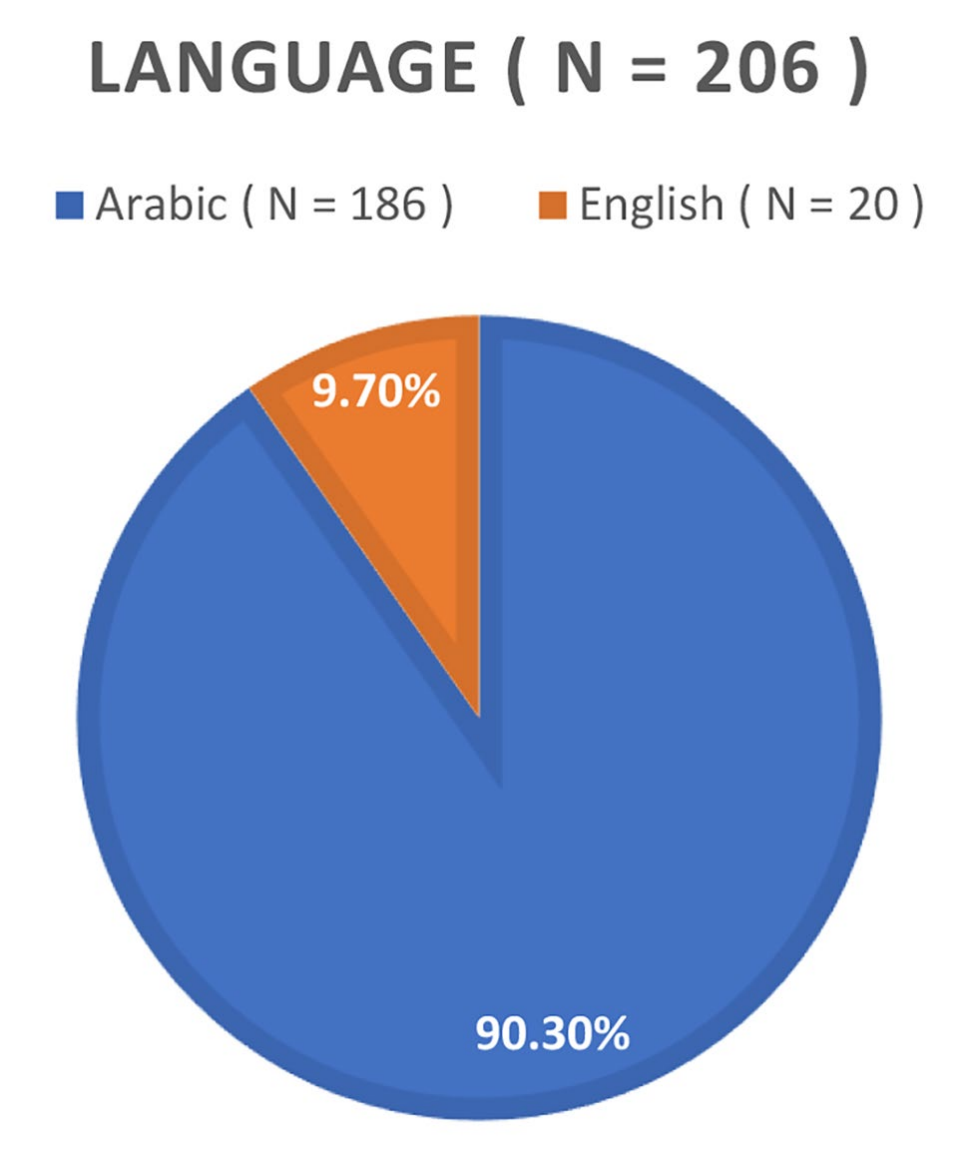
Responses According to language

**Fig. 4:**
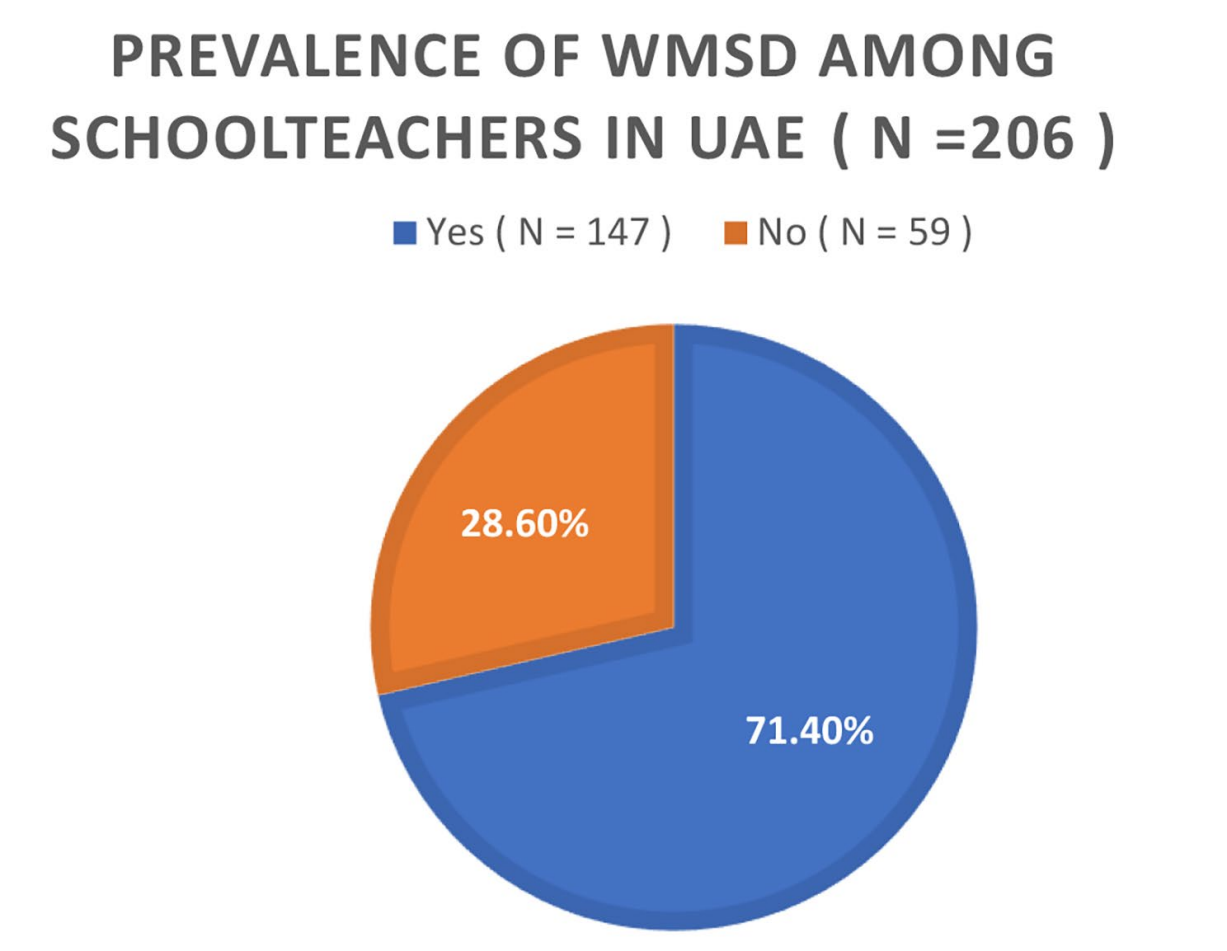
Prevalence of WMSD among schoolteachers in UAE

**Fig. 5:**
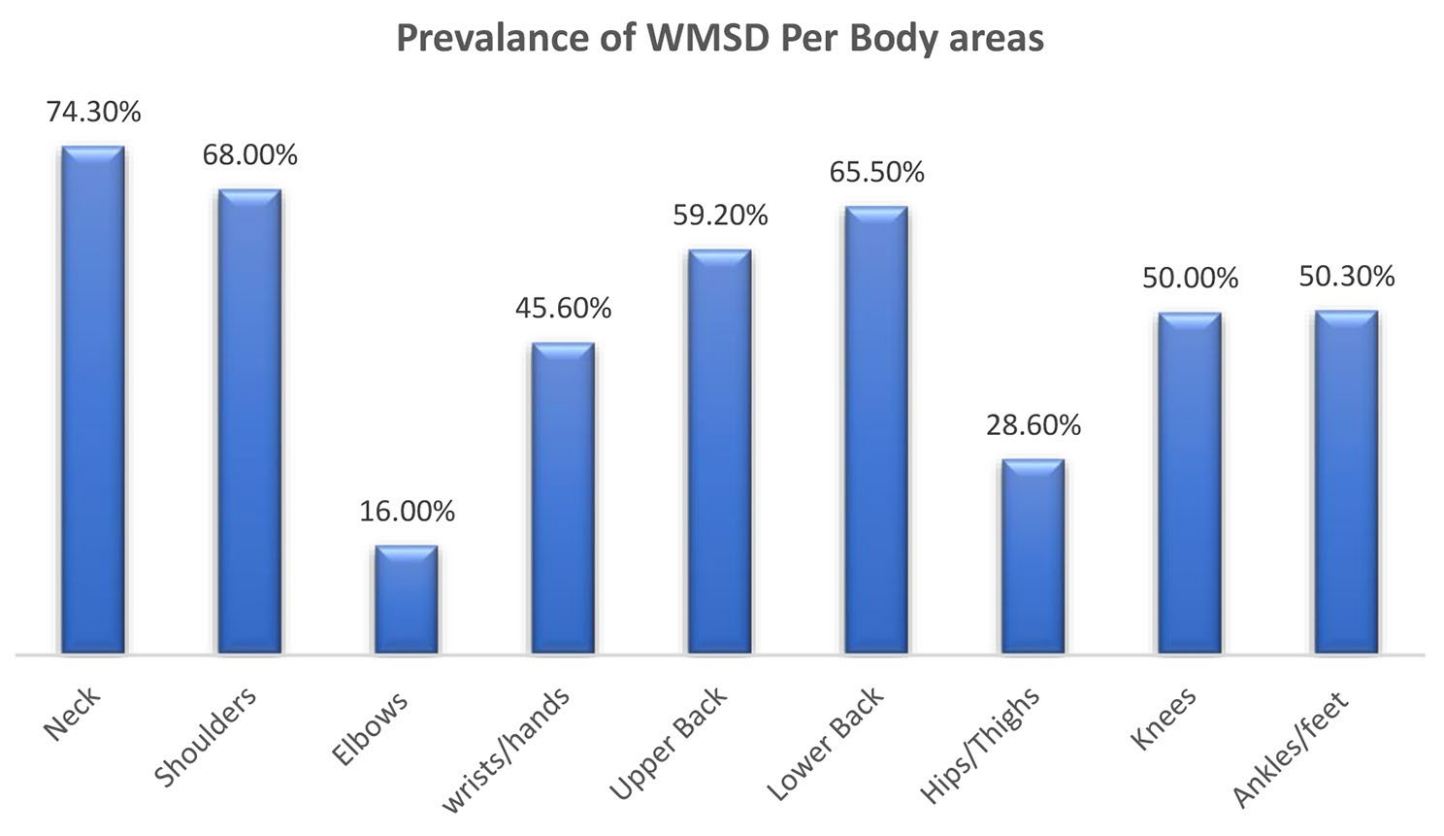
Prevalence of WMSD among schoolteachers per body areas

In Fig. 1, a comprehensive depiction of the study’s participant demographics is provided. Of the total 206 participants, 149 were identified as female, while the remaining 57 were male. While in Fig. 2, Age distribution analysis revealed that within this cohort, 18% of individuals were within the 20 to 30 age range, 26.2% fell within the 30 to 40 age years, 36.9% occupied the 40 to 50 years age brackets, and 18.9% were classified within the 50 to 60 age range. Figure 3 represents the responses according to language, most of the people (around 90%) preferred to respond in Arabic as it is easier for them and it is their native language, while the other 10% could only speaks English as they were not Arabs. The investigation into the prevalence of WMSD among schoolteachers in the UAE yielded noteworthy findings, as depicted in Fig. 4. The overall prevalence of WMSD among the participants was found to be 71.4%. The distribution of WMSD across distinct anatomical regions, it was observed that the neck area exhibited the highest prevalence, with 74.3% of participants reporting discomfort or pain in this region as it is shown in Fig. 5.

Moreover, as it is illustrated in Table 1, the examination of the correlation between WMSD and the level of PA revealed a weak correlation coefficient of 0.142 but statistically significant (*p* value ≤ 0.05).

**Table 1:** Association between Physical Activity (PA) and Work related musculoskeletal disorders (WMSDs) among schoolteachers in United Arab Emirates

Variables (*N* = 206)	Pearson’s Correlation Coefficient (r)	Sig. (*p* value) Two tailed
WMSDs PA	0 0.142	0.042
PA		

*r value represents the correlation coefficient for statical association ranging from − 1 to 1 for a perfect negative and positive association respectively. r value of 0.14 suggests weak correlation

** *p* value of ≤ 0.05 is considered statistically significant

## Discussions

### Overall prevalence

The present study found high prevalence of WMSD among schoolteachers in the UAE, with an overall prevalence rate of 71.4%, underscoring a significant occupational health concern within this population (Graph 4). This finding aligns with a systematic review conducted in 2011, which reported prevalence rates ranging from 39 to 95% for self-reported WMSD among schoolteachers [8]. This high prevalence could also be attributed to several factors such as prolonged static positions, repetitive movements, and emotional stress inherent in the teaching profession. The consequent findings of our study suggest that a considerable proportion of schoolteachers were affected by WMSD, leading to various symptoms such as pain, numbness, tingling, aching, stiffness, or burning. These symptoms can result in work absences, increased work limitations, and potential disability. Additionally, limited breaks and ergonomic challenges could further exacerbate their vulnerability to WMSD.

### Regional prevalence and ergonomic factors with interventional strategies

When examining the prevalence of WMSD in different body segments, the neck area stands out as the most affected among schoolteachers in the UAE, with a prevalence rate of 74.3% (Graph 5). This finding is consistent with several other studies and can be attributed to prolonged “head down” positions during activities such as reading, lesson preparation, and grading, as well as the use of neck extension postures when writing on the board [9–11]. Poor posture, prolonged sitting or standing, and repetitive movements also contribute to neck pain in teachers. Implementing ergonomic interventions and promoting proper posture and movement patterns are crucial for preventing and managing neck related WMSD. In the present study, shoulder area followed the neck as the second most affected segment, with a prevalence rate of 68%. Various studies have reported high prevalence rates of shoulder related WMSD among schoolteachers [12–14]. Overhead activities, carrying heavy bags, and poor ergonomics during teaching activities could contribute to shoulder pain. In contrast, the prevalence of WMSD in the elbows area among schoolteachers in the UAE was relatively lower at 16%. This finding is supported by a recent study conducted in Saudi Arabia which aimed to find the most reported symptoms of WMSD in 12 months among 404 schoolteachers [15]. However, the specific reasons for this lower prevalence require further investigation, and it could be attributed to the nature of teaching tasks that involve fewer repetitive or forceful movements involving the elbows. Wrist/hand related WMSD were prevalent among schoolteachers in the UAE, with a prevalence rate of 45.6%. A study conducted in Kuala Lumpur highlighted the wrist joint as the most affected area [16]. Prolonged writing, computer use, and repetitive hand movements during teaching activities contribute to wrist and hand pain supporting higher prevalence. Moving on to the back segments, WMSD in the upper back area was prevalent among schoolteachers, with a prevalence rate of 59.20%, while in the lower back area it was 65.5%. Similar findings have been reported in studies conducted in Turkey, Iran, Taiwan, and Malaysia [13, 17–19]. Poor ergonomics, prolonged sitting, and heavy lifting contribute to low back pain. Implementing ergonomic interventions, promoting Physical Activity, and providing training on proper lifting techniques would be crucial for preventing and managing lower back related WMSD among schoolteachers. The hips/thighs area showed a prevalence rate of 28.6%, which was relatively lower compared to other body segments. In contrast, a study conducted in Kuala Lumpur reported the hips/thighs area as the second most affected site [16]. The study concluded that prolonged standing, poor posture, and repetitive movements could impact hip health. In our study, the knees ranked as the third most affected site for WMSD among schoolteachers, with a prevalence rate of 50%. A study conducted in Cairo, Egypt, highlighted the impact of sustained and improper sitting postures and recurrent twisting on the knees leading to WMSD [10]. In addition, prolonged standing, walking, and improper knee alignment could also contribute to knee pain. Ankles and feet related WMSD has significant prevalence among schoolteachers in UAE, with a prevalence rate of 50.3%. A study conducted in Malaysia reported the highest prevalence of foot pain among all body sites, with a rate of 32.5% [20]. The study concluded that prolonged standing, improper footwear, and walking on hard surfaces contributed to foot pain.

### Association of WMSD and PA

Regarding the association between WMSD and PA, a weak correlation coefficient of 0.142 was found among schoolteachers which (Table 1). The weak positive correlation implies a marginal linear association with statistical significance (*p* value = 0.042) between the two variables. Though the relation is weak statistically, it has high clinical significance. Clinically it can be suggested that improving PA would have some positive impact on WMSD among schoolteachers. Thus, schoolteachers should implement PA programs in their daily routine as a preventive strategy to cope with WMSD. A weaker association also indicates that factors other than PA might exert more influence on the prevalence of WMSD among schoolteachers in the UAE. The correlation was statistically significant (*p* < 0.05) which implies potential clinical interventions, such as tailored Physical Activity programs or ergonomic adjustments, to mitigate WRMD risk in the workplace. However, caution is warranted as correlation does not imply causation, necessitating further research.

### Interventional strategies with modifiable factors

Tailored interventions based on the correlation’s nature could be beneficial for industries prone to WMSD. Effective communication of these findings is crucial for evidence-based interventions and a healthier, safer work environment. While increasing PA may have a slight influence on WMSD, it is essential to acknowledge that ergonomics and individual characteristics, such as weight, gender, age, and level of PA, also play significant roles in the development of WMSD. Additionally, teaching style, technological proficiency, psychosocial well-being, and cultural background play a role. Factors like dominant hand, teaching load, accessibility needs, and training levels are crucial as well. This weak positive correlation also implies comprehensive interventions that target multiple risk factors, including ergonomics, PA promotion, and individual characteristics, are necessary for effective prevention and management of WMSD among schoolteachers [6, 7, 21]. In line with our study, the research conducted on primary school female teachers found that there was significant prevalence of musculoskeletal pain disorders in the back, shoulder, neck, legs, wrist, and elbow joint. The associated risk factors were type of school, age, weight, number of children, and number of teaching years. Although we only studied the association of PA and WMSD, we consider that the above factors would have contributed to the prevalence significantly and could be modified as a part of effective interventional strategy [22]. In contrast, a study conducted on identifying the associated risk factors for WMSD in gas workers concluded that work duration more than 8 h/ day, sleep duration less that 6 h/day and poor exercise behavior were the most important risk factors and should be accounted [23].

### Ergonomics and recommendations

The importance of work ergonomics has been supported by multiple studies under various professions. A study conducted on WMSD among dental students reported the highest prevalence of neck disorders (82%) due to frequent bending movements and concluded that practice of ergonomics should be incorporated in curriculum and profession [24]. In addition, a study was conducted on Iranian nurses which concluded that ergonomic and organizational interventions for fitting the job to the nurses considering demographic/occupational characteristics are highly essential to improve musculoskeletal system health and relieve fatigue [25]. These findings clearly necessitate the role of ergonomics in WMSD, and consequent modification could be suggested among teaching professionals.

Overall, to enhance ergonomics in classroom settings for schoolteachers, several specific recommendations can be implemented. Firstly, it is crucial to provide adjustable furniture, including chairs and desks, to accommodate varying body types and sizes. Additionally, computer monitors should be positioned at eye level to mitigate neck and eye strain, and chairs should offer lumbar support to maintain proper spinal alignment. Adequate placement of keyboards and mice, along with the provision of footrests for those whose feet do not reach the ground, can further promote ergonomic posture. Varied seating options such as standing desks and stability balls should be considered to encourage movement and reduce prolonged periods of sitting. Regular breaks for stretching and movement are essential, and an organized classroom layout can minimize repetitive bending and reaching. Moreover, the use of anti-glare screens on computer monitors, appropriate lighting, and document holders at eye level can contribute to a comfortable and strain-free work environment. Training sessions and workshops on proper ergonomics techniques should be conducted to raise awareness among teachers, ultimately mitigating the risk of WMSD. These interventions can significantly enhance teachers’ well-being, leading to increased productivity, higher job satisfaction, and improved interactions with students. Moreover, a supportive and ergonomic work environment can aid in teacher retention and recruitment, positively impacting the overall school community. Legal compliance, ethical responsibility, and long-term cost savings on healthcare and absenteeism further underscore the value of investing in these interventions, making them a prudent choice for educational institutions. The affected participants should be approached for awareness about their issues individually while maintaining data confidentially. Possible treatment options such as postural corrections and ergonomic modification should be recommended. Appropriate health care should be provided against WMSD.

Apart, adaptation to work ergonomics and working experience could also help to combat WMSD and reduce the odd risks. A study conducted on non-academic workers in Zimbabwe concluded that increased work experience was protective against the low back pain which was highest prevalent among the participants [26]. Though our study population was not similar, it could be recommended that increased working experienced could help the professionals adapt and develop ergonomic strategies to reduce the risk of WMSD.

### Limitations

The study acknowledges certain limitations such cross-sectional design and convenient sampling imposing selection bias and its inability to ascertain the cause-and-effect relationship. In addition, findings from self-reported data which could have subjective bias and opinion and lack of longitudinal follow-up.

## Conclusions

In conclusion, the study has revealed a significant prevalence (71.4%) of WMSD among UAE school teachers, with the neck (74.3%) and shoulders (68%) being notably affected. Significant discomfort was also observed in various regions, including the upper and lower back, wrists/hands, knees, and ankles/feet. The study’s findings indicate that the correlation between WMSD and PA is weak (0.042), implying that other factors play a more significant role in WMSD prevalence. These results underscore the intricate nature of WMSD in schoolteachers, highlighting the imperative for comprehensive interventions to enhance their occupational well-being. Overall, these findings elucidate the multifaceted landscape of MSDs among schoolteachers and emphasize the importance of targeted strategies to mitigate these occupational challenges. Implementing comprehensive interventions for WMSD among schoolteachers can face several practical challenges. These may include budget constraints for ergonomic improvements, resistance to change in established routines, and the need to accommodate diverse individual needs and preferences. Additionally, logistical considerations, such as finding suitable time slots for exercise breaks or providing specialized training, can pose difficulties. Moreover, ensuring consistent adherence to ergonomic practices and wellness programs may require sustained effort and monitoring. Acknowledging these barriers is crucial for developing realistic strategies and tailored interventions that effectively address the multifaceted nature of WMSD among schoolteachers.

## Data Availability

All data generated or analysed during this study is included in this article. The data set is attached as the related file. Specific data can be made available upon request via email to the corresponding author.
